# Genetic heterogeneity and *trans *regulators of gene expression

**DOI:** 10.1186/1753-6561-1-s1-s80

**Published:** 2007-12-18

**Authors:** Laurel A Bastone, Mary E Putt, Thomas R Ten Have, Vivian G Cheung, Richard S Spielman

**Affiliations:** 1 Division of Biostatistics, Department of Epidemiology and Biostatistics, University of Pennsylvania School of Medicine, 423 Guardian Drive, Philadelphia, Pennsylvania 19104, USA; 2 Department of Genetics, University of Pennsylvania School of Medicine, 415 Curie Boulevard, Philadelphia, Pennsylvania 19104, USA; 3 Department of Pediatrics, University of Pennsylvania School of Medicine, Abramson Research Center, 3516 Civic Center Boulevard, Philadelphia, PA 19104, USA; 4 The Children's Hospital of Philadelphia, Abramson Research Center, 3516 Civic Center Boulevard, Philadelphia, Pennsylvania 19104, USA

## Abstract

Heterogeneity poses a challenge to linkage mapping. Here, we apply a latent class extension of Haseman-Elston regression to expression phenotypes with significant evidence of linkage to *trans *regulators in 14 large pedigrees. We test for linkage, accounting for heterogeneity, and classify individual families as "linked" and "unlinked" on the basis of their contribution to the overall evidence of linkage.

## Background

Microarray technology makes it possible to measure the expression levels of thousands of genes simultaneously. The abundance of expression data has prompted the study of natural variation among humans in baseline gene expression [1-3], including an examination of the genetic determination of gene expression. In a number of studies, gene expression has been treated as a quantitative trait and conventional methods for quantitative trait locus (QTL) mapping have been applied [4,5]. Here, we analyze selected expression phenotypes from the Genetic Analysis Workshop 15 (GAW15) Problem 1 data. Quantitative traits, such as expression phenotypes, are often assumed to be determined by multiple loci. Locus heterogeneity poses a challenge for QTL mapping. When assessing the statistical evidence of an expression phenotype with a single marker, we assume there are two types of families: those whose within-family variation is due to segregation of a QTL linked to the marker ("linked" families) and those whose variation is not ("unlinked" families). The presence of the unlinked families potentially reduces the ability of most statistical methods to detect linkage. Presumably, these families are either not segregating for the trait and are, therefore, uninformative for linkage, or the phenotypic variation in those families is explained by segregation of a QTL located elsewhere in the genome.

Here, we use a statistical model that accounts for heterogeneity and apply our method to follow up linkage evidence of *trans *regulators of gene expression. More specifically, we use a latent class extension of Haseman-Elston (H-E) regression developed by Bastone et al. (unpublished work) that accounts for heterogeneity with respect to linkage. This method includes a classification procedure that is used to determine which individual families are segregating and therefore contribute to the overall evidence of linkage.

The materials and data for this study were previously described by Morley et al. [4]. Gene expression values and single-nucleotide polymorphism (SNP) genotypes were obtained from cell lines derived from 14 large CEPH (Centre d'Etude du Polymorphisme Humain) pedigrees. In the original study, Morley et al. employed a genome-wide linkage mapping approach to detect both of the two possible classes of expression regulators: *cis *and *trans *regulators. The 142 expression phenotypes exhibiting linkage evidence that achieved genome-wide significance were further classified as *cis *and *trans*, based on the location of the linked SNP marker relative to that of the expressed gene. Here, we expand upon the findings of Morley et al. [4] using data for the 5 expression phenotypes with the strongest statistical evidence of *trans *linkage (Table 1 of Morley et al. [4]). We refer to these as the "singleton" phenotypes.

**Table 1:** Results of latent class analysis of linkage

			Class 1 "unlinked" families		Class 2 "linked" families	
				
Phenotype	Chromosome with *trans *peak	Marginal (One-class) slope (*p*-value)	Slope	No. of families in Class 1	Slope (*p*-value)	No. of families in Class 2
*ALG6*	19	-0.04 (<0.001)	-0.01	12	-0.17 (<0.001)	2
*CBR1*	15	-0.67 (0.002)	0.12	11	-3.10 (<0.001)	3
*DSCR2*	9	-0.07 (<0.001)	-0.02	11	-0.27 (0.015)	3
*HOMER1*	9	-0.42 (<0.001)	-0.13	11	-1.31 (<0.001)	3
*TNFRSF11A*	3	-0.93 (0.067)	0.08	12	-4.04 (0.001)	2

"Linked" and "unlinked" families were identified for the five expression phenotypes that showed strongest evidence of linkage in *trans*.

In a second analysis, we analyze data for a set of 29 phenotypes mapping to the putative "master regulator" on 14q32 (Figure 2 of Morley et al. [4]), and a set of 24 phenotypes mapping to a second putative master regulator on 20q13. We refer to these as the "chromosome 14", and "chromosome 20" phenotypes, respectively. Because the genes with putative QTLs on chromosome 14 and chromosome 20 are located elsewhere in the genome, they represent a special case of *trans*-regulated phenotypes, grouped together due to the fact that several expression phenotypes mapped to the same 5-Mb window.

## Methods

We used a latent class linkage approach to detect heterogeneity among families in terms of two latent classes, one of which is tested for linkage (i.e., "linked class"), using an extension of Haseman-Elston (H-E) regression. Using an empirical Bayes approach, we then estimated the posterior probability that each of the families is a member of the linked class. On the basis of these probability estimates, we classified each individual family into one of the two latent classes. For the singleton phenotypes, we used the family class assignments to repeat the linkage scan, stratified by family type. The stratified genome scan allows us to ask several questions regarding the linkage evidence. First, we used the results to test our method and support the plausibility of the class assignments. Second, on the basis of these class assignments, we estimated how many families are contributing substantially to the overall evidence of linkage. Finally, we looked for additional QTLs by determining whether the evidence for linkage elsewhere in the genome increases in the remaining families.

### Haseman-Elston regression

Haseman and Elston [6] describe the regression ("H-E regression") that is performed in each of the two latent classes. H-E regression involves regressing a function (Y) of the phenotype values of two siblings on the number of alleles shared identically by descent (IBD) (X) between the two siblings at a marker. We refer to this as the marginal, or one-class, regression model. Here, we use the squared difference as the outcome variable in H-E regression. Several authors discuss the use of other phenotype functions, including mean-corrected products and weighted combinations of squared sums and differences. Because these phenotype functions typically rely on calculating means or residuals based on the marginal model, their use in a latent class model is not straightforward and could potentially introduce bias or decrease the power of the method to detect the latent classes. Therefore, our latent class extension of H-E regression was developed using the squared phenotypic difference as the outcome variable and for the results to be directly comparable, we use the squared difference in the one-class model as well.

We fit the one-class model using generalized estimating equations and a working correlation structure with two correlation parameters for sib pairs that share zero or one individual in common, as implemented in S.A.G.E. [7]. For each expression phenotype, we performed a genome scan (one-class model) in all 14 families.

### Latent class model

Latent class methodology assumes that the distribution of the outcome variable (i.e., phenotype function) is a mixture of two or more parametric distributions. Let *S *be a multinomial variable indicating class membership of a given family, with probability of membership for latent class s of π_*s *_= *P*(*S *= *s*), *s *= {1, 2}. For class *s*, we assume a linear H-E regression model for the squared phenotypic difference, conditional on latent class and IBD sharing at the marker: *E*[*Y*|*X*, *S *= *s*] = α_*s *_+ β_s _*X. We assume the marginal density of the outcome variable is a mixture of the conditional densities within the latent classes, such that the marginal mean is *E*[*Y*|*X*]= ∑_*s *_π_*s *_(α_*s *_+ β_s _*X). *Y *is regressed on *X *within each class, and a test for linkage is based upon the magnitude of the regression slope in the segregating class. The null hypothesis that the regression slope in this class equals zero, is tested against the one-sided alternative that the regression slope was less than zero. Empirical Bayes estimates of the parameters in the latent class model were used in turn to estimated the posterior probability that a given family is in latent class *s*, *P*(*S *= *s*|*Y*, *X*). We estimated the probability of membership in the "linked" class for each family and subsequently assign families to their more likely class.

Based on the results of the one-class model, we select the single SNP exhibiting the maximum evidence of linkage with the trait and obtain multipoint IBD sharing information using Merlin [8]. The raw phenotype values were converted to squared differences for all possible sib pairs per family. The parameters of the latent class model were estimated using the maximum likelihood method. Maximization was carried out using a general quasi-Newton procedure implemented by SAS PROC TRAJ (v.8) using the NOVAR option to omit calculation of standard error estimates [9]. The genetic hypothesis of linkage requires an additional assessment of a significant negative slope in one of the latent classes ("linked" class). The significance of the class-specific slope in the linked class was tested by permuting the IBD sharing within each family, re-fitting the latent class model, and comparing the minimum slope in the original data to that in each set of permuted data. Our permutation approach preserves both the clustering by family and any potential correlations among sib pairs within a family.

## Results

### Primary analysis

Evidence of heterogeneity was detected under the linkage model for all five singleton phenotypes. More specifically, in the two-class model, all phenotypes exhibited evidence of a zero slope in one class, and a statistically significant negative slope in the other ("linked") class (See Table 1). For all phenotypes, the estimate of the slope in the "linked" class is more negative than that of the "marginal" slope (i.e., the slope obtained in the one-class model). As one would expect, the marginal slope appears to be an average of the negative and zero slope in the two classes.

We assigned each family to its more likely class, on the basis of estimates of family-specific probabilities of latent class membership. In general, the classes were well separated, with estimates of class membership probabilities close to zero and one. For families classified as "linked", the estimates of the probability of membership in the "linked" class ranged from 0.918 to 1.000, with a mean of 0.988. For families classified as "unlinked", the estimates ranged from 0.000 to 0.024, with a mean of 0.001. We found that relatively few families (i.e., two or three families) contribute to the overall evidence of linkage, despite the strong marginal effect. This suggests that for these five phenotypes, we detected QTLs with relatively rare alleles with strong effects.

**Figure 1:**
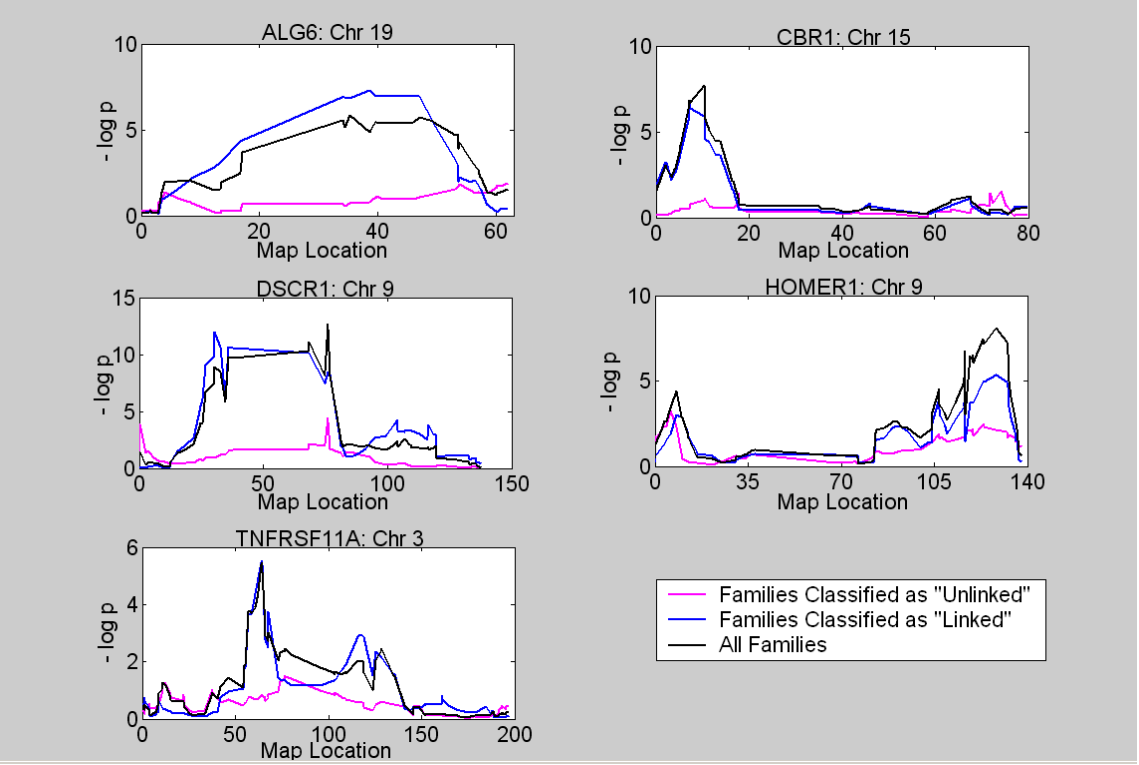
**Linkage scans stratified by family type**. Families were classified as "unlinked" or "linked" and class-specific linkage scans were carried out only for the chromosome on which the strongest evidence for linkage in *trans *was found.

We repeated the linkage scans for each class separately. Figure 1 shows the full-sample and class-specific linkage scans for all five phenotypes, using markers on the *trans *peak chromosome. The stratified scans were implemented in S.A.G.E. [7]. These graphs suggest that we can effectively use the latent class model to identify the subset of families that provide most of the evidence of linkage.

In addition to the chromosome-specific linkage scan, we performed a stratified, genome-wide linkage scan for each singleton phenotype. By omitting those families whose phenotypic variation is already explained ("linked" families above), we might have more power to detect additional QTLs. This reasoning assumes that, for example, when families in the sample are segregating for two distinct QTLs, individual families will be segregating for one or the other, but not both QTLs. Of course, this is not necessarily true. However, if the less common allele at each of the QTLs is rare, it may be a good working assumption. In the linkage scans, the evidence for linkage is measured by the negative log *p*-value from H-E regression. The maximum new negative log *p*-value in "unlinked" families ranged from 3.3 for *CBR1 *(increased from 0.3 in all families) to 4.1 for *DSCR2 *(increased from 2.2 in all families). In the original analysis, Morley et al. [4] used two different levels of stringency for determining the genome-wide significance of evidence for linkage. A negative log *p*-value of 4.4 was used as a less stringent threshold. Thus, while the evidence of linkage to other regions in the genome did increase in the "unlinked" families, none of the new linkage peaks achieved genome-wide significance by this criterion.

### Secondary analysis

We next applied the latent class method to the chromosome 14 and 20 phenotypes. Specifically, we analyzed data for 29 phenotypes with significant evidence of linkage to a 5-Mb region on chromosome 14. Again, we found that a relatively small number of families contributed to the overall evidence of linkage (range 1 to 7, average 2.4). In fact, for 12 phenotypes, only one family is classified as "linked". Surprisingly, a single family (CEPH 1418) is classified as "linked" for all phenotypes. We repeated a linkage scan removing that family and found that only one phenotype continued to exhibit linkage to chromosome 14. We compared the expression values of Family 1418 to those of the other families and found the mean and range of expression values to be quite similar. Thus, there is nothing remarkable about this family with respect to phenotypic values. The strong dependence of the linkage findings on this family might indicate a relatively rare allele at a QTL with a very strong effect, detected primarily in one family.

We repeated the analysis described above for 24 expression phenotypes with significant linkage to a 5-Mb region on chromosome 20. In contrast to the results for the chromosome 14 phenotypes, we found that different families contribute linkage evidence for different phenotypes. All families contribute evidence for at least two phenotypes and the maximum number of phenotypes to which one family contributes is 14. In general, somewhat larger numbers of families contributed to the evidence of linkage for the chromosome 20 phenotypes (range 1 to 9, average 3.6), as compared to the chromosome 14 phenotypes.

## Conclusion

We applied a latent class extension of H-E regression to evaluate heterogeneity and linkage of expression phenotypes to *trans *regulators of gene expression. The family classification procedure under the latent class model allows us to estimate which families are contributing to the overall evidence of linkage. A key feature of the latent class approach is that it provides a means for classifying families by fitting a single model. One could imagine an alternative approach in which the linkage analysis was repeated for all possible combinations of families in order to find the subset of families for which the evidence of linkage was strongest. However, this alternative approach would introduce a substantial multiple-testing problem that is avoided by our unified latent class approach.

We used the family class assignments that follow from the latent class model to estimate the number of families contributing to the overall evidence for linkage. We also used the procedure to identify influential families ("linked"), thereby identifying a subset of families ("unlinked") in which to look for additional QTLs. For all five phenotypes considered here, we found that a small number of families were classified as "linked". The fact that for those genes, significant evidence of linkage was found in the one-class model is likely due to the large sibship size in the CEPH families and the fact that alleles at QTLs regulating gene expression may have relatively large effects. We applied the latent class model to target genes that were pre-selected based on the results of Morley et al. [4]. Determining under what conditions the latent class model may have more power to detect linkage in the absence of significant evidence of linkage in the marginal model is a topic of future research. In sets of data with substantially smaller sibships (e.g., two to four offspring per family), our latent class model can be applied, but it is likely that larger sample sizes (more families) will be required in order to have adequate power to detect linkage.

Linkage between expression phenotypes and putative *cis *and *trans *regulators has been detected in various studies. In a number of cases, the linkage findings for *cis *regulators have been supported by subsequent analyses. For example, Morley et al. [4] found strong evidence of association between SNP markers and expression phenotypes that have evidence of linkage to a *cis *regulator. In contrast, validating evidence of linkage to *trans *regulators by association methods has been difficult and the genetic dissection of *trans *regulators remains a challenge. It is likely that genetic heterogeneity contributes to the difficulty of identifying *trans *regulators. Methods such as the one applied in this study that appropriately model genetic heterogeneity may improve our ability to map *trans *regulators and, ultimately, increase our understanding of the genetics of gene expression.

## Competing interests

The author(s) declare that they have no competing interests.
